# A lung cancer risk classifier comprising genome maintenance genes measured in normal bronchial epithelial cells

**DOI:** 10.1186/s12885-017-3287-4

**Published:** 2017-05-02

**Authors:** Jiyoun Yeo, Erin L. Crawford, Xiaolu Zhang, Sadik Khuder, Tian Chen, Albert Levin, Thomas M. Blomquist, James C. Willey

**Affiliations:** 1 Division of Pulmonary and Critical Care Medicine, Department of Medicine, The University of Toledo College of Medicine, 3000 Arlington Avenue, HEB 219, Toledo, OH 43614 USA; 2 Cancer Genetics and Comparative Genomics Branch (CGCGB), National Human Genomes Research Institute (NHGRI), National Institutes of Health (NIH), Bldg 50, Rm 5341, 50 South Dr., Bethesda, MD 20892 USA; 3 Division of Pulmonary and Critical Care Medicine, Department of Medicine, The University of Toledo College of Medicine, 3000 Arlington Avenue, RHC 0012, Toledo, OH 43614 USA; 4 Department of Mathematics and Statistics, The University of Toledo, 2801 W. Bancroft Street, Toledo, OH 43606 USA; 5 Department of Biostatistics, Henry Ford Health System, 1 Ford Place, Detroit, MI 48202 USA; 6 Department of Pathology, The University of Toledo College of Medicine, 3000 Arlington Avenue, Toledo, OH 43614 USA; 7 Ruppert 0012, Division of Pulmonary and Critical Care Medicine, Department of Medicine, The University of Toledo College of Medicine, 3000 Arlington Avenue, Toledo, OH 43614 USA

**Keywords:** Lung cancer risk, Lung cancer screening, Low dose helical CT screening, Lung cancer risk test biomarker, Genome maintenance, DNA repair, Antioxidant enzymes, Bronchial epithelial cells

## Abstract

**Background:**

Annual low dose CT (LDCT) screening of individuals at high demographic risk reduces lung cancer mortality by more than 20%. However, subjects selected for screening based on demographic criteria typically have less than a 10% lifetime risk for lung cancer. Thus, there is need for a biomarker that better stratifies subjects for LDCT screening. Toward this goal, we previously reported a lung cancer risk test (LCRT) biomarker comprising 14 genome-maintenance (GM) pathway genes measured in normal bronchial epithelial cells (NBEC) that accurately classified cancer (CA) from non-cancer (NC) subjects. The primary goal of the studies reported here was to optimize the LCRT biomarker for high specificity and ease of clinical implementation.

**Methods:**

Targeted competitive multiplex PCR amplicon libraries were prepared for next generation sequencing (NGS) analysis of transcript abundance at 68 sites among 33 GM target genes in NBEC specimens collected from a retrospective cohort of 120 subjects, including 61 CA cases and 59 NC controls. Genes were selected for analysis based on contribution to the previously reported LCRT biomarker and/or prior evidence for association with lung cancer risk. Linear discriminant analysis was used to identify the most accurate classifier suitable to stratify subjects for screening.

**Results:**

After cross-validation, a model comprising expression values from 12 genes (CDKN1A, E2F1, ERCC1, ERCC4, ERCC5, GPX1, GSTP1, KEAP1, RB1, TP53, TP63, and XRCC1) and demographic factors age, gender, and pack-years smoking, had Receiver Operator Characteristic area under the curve (ROC AUC) of 0.975 (95% CI: 0.96–0.99). The overall classification accuracy was 93% (95% CI 88%–98%) with sensitivity 93.1%, specificity 92.9%, positive predictive value 93.1% and negative predictive value 93%. The ROC AUC for this classifier was significantly better (*p* < 0.0001) than the best model comprising demographic features alone.

**Conclusions:**

The LCRT biomarker reported here displayed high accuracy and ease of implementation on a high throughput, quality-controlled targeted NGS platform. As such, it is optimized for clinical validation in specimens from the ongoing LCRT blinded prospective cohort study. Following validation, the biomarker is expected to have clinical utility by better stratifying subjects for annual lung cancer screening compared to current demographic criteria alone.

**Electronic supplementary material:**

The online version of this article (doi:10.1186/s12885-017-3287-4) contains supplementary material, which is available to authorized users.

## Background

Lung cancer kills nearly 160,000 people per year in the United States, more than breast, prostate, and colon combined [1]. Importantly, high risk subjects (age 55–80, 30 or more pack-years smoking, and quit smoking <15 years prior) screened annually with low dose CT (LDCT) were more likely to be diagnosed in early stage and this was associated with a 20% reduction in mortality in the National Lung Screening Trial (NLST) [2, 3]. Based on these findings, annual LDCT screening is now recommended for high risk subjects by the United States Preventive Safety Task Force (USPSTF) [4]. While the benefits of screening are clear, they are limited due to large variation in risk among those eligible by demographic criteria alone [5–9]. The screen-eligible group is estimated to comprise 5 and 10 million individuals [3, 10]. However, less than 10% of individuals at risk based on these demographic criteria are expected to develop lung cancer in their lifetime. As highlighted in the 2014 USPSTF summary statement, there is a clear and well-recognized unmet need for development of biomarkers that can define lung cancer risk better than demographic criteria alone and enable screening efforts to focus on persons who are at highest risk [11].

Inter-individual variation in lung cancer risk is in part determined by variation in function of normal bronchial epithelial cells (NBEC) that form the bronchial epithelium. This epithelial layer represents a barrier that protects the lung functional units and the rest of the body from environmental and occupational air-born hazards including cigarette smoke, radon gas, asbestos, and ozone. NBEC are specialized to express many antioxidant, DNA repair, and xenobiotic metabolism enzyme genes at high level [12, 13]. Importantly, many key genome maintenance (GM) gene pathways, particularly those responsible for antioxidant protection (AO), DNA repair (DNAR), and/or stem cell regenerative potential are regulated differently in normal lung tissue of lung cancer subjects compared to controls [14–17].

These differences in GM pathway gene regulation in NBEC of cancer subjects may be partly due to inherited germ-line variation in NBEC gene regulation and/or acquired effects. Evidence for the role of *inherited pre-disposition *in the form of germ-line variation in GM regulation comes from both GWAS and experimental studies. For example, in a recent GWAS meta-analysis, putative *cis*-regulatory variants in GM genes in the G2/M DNA damage checkpoint and DNA repair pathways were were associated with risk for lung cancer [18]. In recent experimental studies, DNA variants were identified that are responsible for inter-individual variation in NBEC regulation of the key nucleotide excision repair gene ERCC5 and a key transcription regulator of GM genes, CEBPG [19–21]. Evidence for the role of *acquired NBEC changes* in lung cancer risk includes analysis of gene expression and somatic genetic mutations in NBEC. For example, certain patterns of gene expression in NBEC are characteristic of effects from heavy smoking [22, 23]. In addition, acquired effects from cigarette smoke create a molecular field of injury in the airway epithelium that may represent an early stage of carcinogenesis [24–26]. A field of injury may include morphologic changes that are preceded and/or accompanied by somatic mutations, epigenetic modifications, and metaplastic differentiation. NBEC transcript abundance patterns associated with the presence of early lung cancer have been described and classifiers based on these findings currently are being evaluated as biomarkers to guide diagnostic testing [27, 28].

We previously reported that a lung cancer risk test (LCRT) classfier comprising 14 genes measured in NBEC accurately classifies cancer (CA) from non-cancer (NC) subjects [14]. This classifier includes key NBEC GM genes in AO, DNAR, and cell cycle control (CCC) pathways. We hypothesis that the association of this classifier with lung cancer is largely, if not entirely, due to inherited DNA variants responsible for sub-optimal regulation of GM genes in NBEC. In an effort to further optimize this biomarker for increased specificity and ease of clinical implementation, we used a recently developed targeted competitive multiplex PCR amplicon library method [29] for RNAseq to measure high prior likelihood GM pathway gene targets in NBEC specimens from a retrospective cohort of 120 subjects, including 61 CA cases and 59 NC controls. The overall goal is to develop a biomarker that will identify individuals who meet current eligibility criteria for annual LDCT screening but have such low risk based on LCRT biomarker measurement that they can be safely advised to opt out of screening. After optimization of the biomarker reported here, it will be used to assess NBEC samples from the prospective LCRT cohort [30].

## Methods

### Study subjects and bio-specimens

NBEC specimens analyzed were from a retrospective cohort of 120 subjects, including 61 CA cases and 59 NC controls. The controls were confirmed to not have lung cancer at time of sample collection based on negative imaging, bronchoscopy, and follow-up according to standard of care. NBEC specimens were obtained from each subject by cytology brush biopsy of grossly normal appearing main stem bronchi from subjects at the University of Toledo according to previously described methods [29]. Collection and use of these samples and corresponding medical/demographic data was approved under UT IRB protocols #108538 and #107844. Each subject included in this study provided written informed consent.

### RNA extraction

RNA was extracted from NBEC using TRI reagent (Molecular Research Center, Cincinnati, OH). RNA was reverse transcribed into cDNA with M-MLV reverse transcriptase (Invitrogen, Carlsbad, CA) using oligo dT primer according to the manufacturer’s protocol as previously reported [12]. Most RNA samples were processed to cDNA immediately and cDNA was stored at −20 °C. In the few cases when samples were stored as RNA prior to reverse transcription (RT), they were stored under EtOH at −80 °C.

### RNA quality assessment

Presence of gDNA contamination in RNA samples was assessed by two methods. First, the presence of genomic material in NBEC cDNA was quantified in a representative set of samples using commercially available SCGB1A1 genomic DNA reagents (Accugenomics, Inc., Wilmington, NC) as previously described [20]. Second, for the majority of targeted region assays the reagents were designed to span introns. Thus, if there were significant gDNA contamination of the RNA and this carried over to cDNA following RT, PCR would yield larger product bands for these targets after electrophoresis. RNA integrity by comparison of cDNA yield at two or more regions with varying distances from the 3′ end of the transcript, assuming that if the transcript was degraded this would be associated with breaks at intervals and there would be lower representation at the more 5′ location.

### Target selection

The 68 analytes selected include alternative transcripts expressed by a sub-set of genes in the previously reported classifier [14], as well as an expanded list of AO, DNAR, and CCC pathway genes with high prior likelihood for involvement in lung cancer risk based on studies from this laboratory and others. (Additional file 1: Table S1.

### Targeted RNAseq analysis

We used a targeted competitive multiplex PCR amplicon library method developed in this laboratory, comprising first-round competitive *multiplex* PCR pre-amplification with second round *low-complexity* amplification. With this NGS library preparation method, multiple target analytes were simultaneously PCR-amplified in a reaction mixture containing a known number of competitive internal standard (IS) molecules for each respective analyte. Because each pair of analyte native template (NT) and respective competitive IS contained the same primer sequences and was amplified with the same efficiency, the starting analyte copy number could be calculated as the product of a) number of IS molecules in the starting PCR reaction times, b) the ratio of analyte sequence reads/internal standard sequence reads following PCR [31]. This approach ensured quality control for a) analyte copies loaded into library, and b) loading of amplicon products into sequencer [32]. Control at each of these levels is key to avoiding analytical variation due to stochastic sampling. Further, this approach enabled reliable quantification of each analyte, while promoting convergence of products during PCR. This convergence of products markedly reduced sequencing space required for analysis [29, 32, 33].

### Competitive multiplex PCR amplicon library preparation

A targeted competitive multiplex PCR amplicon library was prepared for each sample to quantify expression of the 68 assays representing 33 selected GM genes.

### Primers

We designed a pool of forward and reverse primer sets targeting the selected analytes (Additional file 1: Table S1). For each analyte assay, primers were designed to target 101-bp regions and were synthesized by Integrated DNA Technologies (IDT, Coralville, Iowa, USA). A universal tail sequence (arrayed primer extension: APEX-2 [34]) that is not present in the human genome was added to the 5′ end of each analyte-specific gene primer and served as a linker for the next step, the index barcoding PCR. For barcoding PCR, primers were designed with APEX sequence at the 3’end (homologous to the 5′ end of the analyte-specific primers), barcode sequences in the middle, and Illumina read1 or read2 sequences at the 5’end. A unique combination of forward and reverse barcodes was selected from the available pool to identify each sample. The 5′ read 1 and read 2 sequences in the barcoding primers served as linkers for the last step PCR that added a platform specific adaptor, in study presented here for the Illumina sequencing flow cell.

### Preparation of internal standard mixture (ISM)

For each analyte assay, we designed an IS that had 6 base pairs altered compared to the reference DNA (NCBI, GRCh37) and all IS were then batch synthesized (CustomArray Inc., Bothell, WA, USA). To create full-length, double-stranded products, we used assay-specific primers to PCR-amplify each IS from the batch product. PCR products were quantified, mixed and gel-purified to create an internal standards mixture (ISM) for use in PCR. The ISM contained 10^−15^ M targets (600 copies/μL) relative to the 10^−14^ M ACTB IS (6000 copies/μL).

### PCR 1

For each reaction, a 10 μL reaction volume was prepared containing 1 μL cDNA sample, 1 μL ISM (final amount in reaction 600 copies target IS/6000 copies ACTB IS), 1 μL 68-plex primer pool containing target-specific forward (F) and reverse (R) primers with APEX-2 linker tail (100 nM each), 1 μL of 2 mM dNTPs, 1 μL of 10× Idaho Technology reaction buffer with 30 mM MgCl_2_, 0.1 μL of Promega GoTaq Hot Start Taq polymerase (5 U/uL), and 4.9 μL of RNase-free water. Each reaction mixture was amplified using an air thermal cycler (RapidCycler; Idaho Technology, Inc. Idaho Falls, Idaho). After an initial Taq activation at 95 °C for 3 min, the reaction mixture was run for 20 cycles, with each cycle consisting of 94 °C for 5 s (denaturing), 58 °C for 10 s (annealing), and 72 °C for 15 s (extension).

### PCR 2: Microfluidic PCR

The PCR 1 product for each sample was column-purified (50uL elution, QIAquick® PCR Purification Kit, Qiagen, Valencia, CA) to remove residual primers and salts. Next, 1 μL of purified PCR 1 product was loaded into one of 48 wells of a Fluidigm AccessArray™ chip (Fluidigm, South San Francisco, CA). Primer pairs for one or two assays were loaded into each of the 48 primer wells on the chip. Each combination of 48 samples and 48 wells of primers (total 70 sets of primers) was automatically combined with PCR reaction mixture on the AccessArray, then PCR-amplified in the microfluidic chambers according to AccessArray™ protocol. The 48-PCR products for each sample were harvested into each sample well. After completion of PCR, we checked PCR products randomly on an Agilent Bioanalyzer 2100 (Santa Clara, CA) to ensure presence of correct size PCR products.

### PCR 3: Barcoding PCR

The PCR 2 product for each sample was column-purified (50uL elution, QIAquick® PCR Purification Kit) to remove residual primers and salts. One microliter of purified product from each sample was used for barcoding PCR. A 10 μL PCR reaction volume was prepared containing: 1 μL of purified PCR 2 product, 1 μL of 10 μM each sample specific forward and reverse barcoding primer, 1 μL of 2 mM dNTPs, 1 μL of 10× Idaho Technology reaction buffer with 30 mM MgCl_2_, 0.1 μL of Promega GoTaq Hot Start Taq polymerase (5 u/μL) and 4.9 μL of RNase-free water. Barcoding of each sample was done with an air thermal cycler under the following conditions: 95 °C for 3 min (Taq activation); 15 cycles of 94 °C for 5 s (denaturation), 58 °C for 10 s (annealing), and 72 °C for 15 s (extension). We randomly checked PCR products on the Bioanalyzer 2100 to ensure presence of correct size PCR products.

### PCR 4

The barcoded PCR 3 product for each sample was diluted (100-fold in 10 mM Tris-Cl, 0.1 mM EDTA (TE) and PCR was conducted to add the platform-specific sequences. For each platform specific PCR reaction, a 10 μL reaction volume was prepared containing: 1 μL of 100-fold diluted barcoding PCR product with TE, 1 μL of 10 μM P5 and P7 primer mixture each, 1 μL of 2 mM dNTPs, 1 μL of 10× Idaho Technology reaction buffer with 30 mM MgCl_2_, 0.1 μL of Promega GoTaq Hot Start Taq polymerase (5u/μL) and 5.9 μL of RNase-free water. Each sample was cycled separately using the same PCR condition as the barcoding reactions. After checking peaks of PCR products and concentration randomly, products from all 120 bio-specimens were combined at equal volumes and then purified with the QIAquick® PCR purification kit. The library concentration was ascertained on the Bioanalyzer 2100 and then the aliquot of library was sent for sequencing.

### Sequencing

Each combined sample library generated in PCR 4 was analyzed at the University of Michigan (UM) Genomics Core Facility on Illumina Hiseq 2500 with TruSeq SBS Kit v4. Following analysis, the UM service center provided raw sequencing data in FASTQ format that were analyzed by our custom pipeline [29, 32]. First, Read 1 and Read 2 sequence were joined and then de-multiplexed based on dual-index barcoding on each template. Then, the locus was identified based on the region of the primer sequences. The “captured” region between the primer sequences was aligned using custom alignment with Approximate String matching algorithm as previous described [29]. The “counts” of alignment for each allele at each locus was provided as an Excel file.

### Filtering for stochastic sampling error and total expression measurement

To avoid stochastic sampling error and sequencing error we established thresholds for minimum molecules loaded into library preparation and minimum number of sequencing read counts, then filtered out data below thresholds as previously described [32]. For the results presented, we used thresholds associated with coefficient of variation (CV) based on stochastic sampling predicted to be <1.0:

$$ CV=-1+{10}^{\left( Molecules\ {Input}^{-0.54}+ Sequence\ {Reads}^{-0.54}-{\left[ Molecules\  Input\  X\  Sequence\  Reads\right]}^{-0.54}\right)} $$

Calculation of analyte NT molecules in 1 μL of cDNA for each target and reference analyte was based on comparison of NT signal (sequence read count) to signal for a known number of input IS. Specifically, for each analyte, NT/IS read counts were compared with the known molecules of input IS, which was 600 molecules for each target analyte and 6000 molecules for ACTB reference analyte. Then the transcript abundance value was calculated for each target and reported as target gene molecules/10^6^ ACTB molecules) (Additional file 1: Table S1).

### Statistical analysis

All transcript abundance values (in molecules/10^6^ ACTB molecules) were log_10_ transformed prior to further analysis. Statistical analyses, including cancer vs. control differences in linear discriminant analysis (LDA) were performed using R (v 3.2.5) (http://www.R-project.org). Twenty-three assays with more than 30% missing values due to low counts and/or sequencing reads were excluded from the analysis leaving 45 transcript abundance assays for building of model by linear discriminant analysis (LDA) (see Additional file 1: Table S1). These transcript abundance assays and three demographic variables, age, gender, and smoking (pack-years) were included in model building.

### Imputation of missing values

Missing assays were imputed using Multivariate Imputation by Chained Equations (MICE) [35] with Predictive Mean Matching (PMM) [36, 37]. MICE is a multiple imputation (MI) technique and can take into account the uncertainty in the imputations and yield accurate standard errors compared with single imputation. PMM is a very popular semi-parametric method within the MI framework. It imputes missing values from nearest-neighbors, where distance is based on the expectation of the missing variables conditional on the observed covariates**.** M = 50 copies of the data were created, each of which had the missing values suitably imputed using the MICE procedure, resulting in M multiply imputed datasets. Each complete dataset was analyzed independently and identically.

### Building of classifier model

#### Feature selection in complete dataset

For each complete data set, correlation-adjusted-t (CAT) scores [38] were used to calculate the overall ranking for each feature. CAT analysis takes the feature-feature correlations into consideration and can be viewed as “decorrelated” t-score. Thus it measures the individual contribution of each feature to separate CA and NC patients after removing the effects of all other features and provides a natural feature ranking criterion and facilitates the feature selection.

#### Feature selection in multiply imputed data

Each imputed data set will typically result in LDA models with different selected features. The LDA model was built on each copy of imputed data set using features with at least 60% of “votes”. Area under the curve (AUC) and classification accuracies were averaged across the M copies to give a single summary value. The best set of features was considered to be those comprised by the LDA model with the highest average AUC with 10-fold cross-validation. Standard errors of the average were computed according to the “Rubin rules” [39]. The Receiver Operator Characteristic (ROC) curve was plotted and error calculated by R (v 3.2.5).

## Results

### NBEC RNA sample characteristics

In each of the representative samples assessed, the signal for genomic copies, using the genomic DNA-specific SCGB1A1 reagents (Accugenomics, Inc., Wilmington, NC) [20] was below the threshold for detection (< 6 copies in cDNA containing 60,000 copies ACTB. Following PCR of cDNA using target reagents that crossed introns and electrophoresis of PCR products, the electropherograms were free of gDNA product bands. High RNA integrity was demonstrated by the high yield of PCR products for target regions at both the 5′ and 3′ end of transcripts for all genes in which multiple regions were targeted (19 of the 33 genes assessed).

### Cohort characteristics

After filtering measured values to remove those that did not meet the threshold for molecules loaded into library preparation and PCR product loaded into sequencer (i.e. sequencing read counts), we removed five subjects for whom there were results for <6 assays/subject. Following this, there were 115 subjects (58 CA and 57 NC controls).

The CA group was significantly different from NC group with respect to Age (*p* = 0.022), gender (*p* = 0.021), and smoking history (*p* = 0.035), but not pack-years (*p* = 0.072) (summary statistics are provided in Table 1 **and details for each subject in** Additional file 1: Table S1. Among the 106 subjects for whom ethnicity was known, the cohort was 86% non-Hispanic white, 12% African American, and 2% Asian or Hispanic.

**Table 1:** Demographic characteristics of the study population

	**Non-cancer (** ***n*** **= 57)** ^**a**^	**Cancer (** ***n*** **= 58)** ^**a**^	***p*** **-value** ^**b**^
Age, yr	59.3 (±14.2)	64.4 (±9.5)	*0.022*
Gender			*0.021*
Male	28	40	
Female	29	16	
Smoking history			*0.035*
Current	23	12	
Former	27	37	
Never	0	1	
Pack-Years	43 (±28.7)	53 (±31.3)	*0.072*
Ethnicity			
White	45	46	
AA	9	4	
Other	1	1	

^a^Missing data: age (*n* = 2), gender (*n* = 2), smoking history (*n* = 15), ethnicity (*n* = 9). ^b^ *p*-values were calculated using a Student’s t-test for age and pack-years, and Fisher exact test for gender and smoking history. AA: African American.

### Linear discriminant analysis (LDA)

**Fig. 1:**
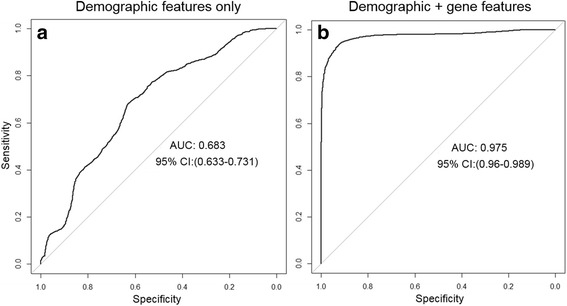
Receiver operating characteristic curve (ROC) for performance of best classifier in 57 NC and 58 CA subject based on M = 50 multiple imputations

We used LDA to assess each of 48 features for CA vs NC classification accuracy, including 45 transcript abundance assays and the three demographic variables, age, gender, and pack-years smoking. We first calculated the CAT score for each feature and then identified the best model using 10-fold cross-validation. The best classifier included transcript abundance values for 13 assays located on 12 genes. The list of transcript abundance features in the best classifier, with function and frequency of contribution to classifier during model development is provided in Table 2 and information for all 48 features used in model building are presented in Additional file 1: Table S1. The best classifier had ROC AUC 0.975 (95% CI: 0.96–0.99) (Fig. 1) and overall classification accuracy 93% (95% CI 88%–98%) (Table 3). This classifier was significantly (*p* < 0.0001) more accurate than the best model comprising demographic features only (Fig. 1, Table 3). With the threshold set at 95% sensitivity, as might be applied for screening, the specificity was 90%.

**Table 2:** Classifier feature characteristics

Feature	Function	Ranking	Selection frequency	% Missing value
E2F1	CCC/DNAR	1	1	23
ERCC5	DNAR	2	1	9
XRCC1	DNAR	3	0.94	8
GPX1	AO	4	0.92	2
TP63–2	CCC/DNAR	5	0.92	30
GSTP1	AO	6	0.88	12
CDKN1A	CCC/DNAR	7	0.86	2
TP53–2	CCC/DNAR	8	0.86	9
ERCC4–2	DNAR	9	0.78	29
RB1	CCC/DNAR	10	0.74	23
ERCC5–2	DNAR	11	0.70	20
KEAP1–2	AO	12	0.70	11
ERCC1–2	DNAR	13	0.68	4

*AO* antioxidant protection, *DNAR* DNA repair *CCC* cell cycle control

**Table 3:** Classifier Performance

	**Demographics only**	**Demographics plus genes**
Number of subjects (N)	115 (NC:57, CA:58)	115 (NC:57, CA:58)
Demographic variable used	3: gender, age and pack-years	3: gender, age and pack-years
Picked Gene Features	0	E2F1 ERCC5 XRCC1 GPX1 TP63.2 GSTP1 CDKN1A TP53.2 ERCC4.2 RB1 ERCC5.2 KEAP1.2 ERCC1.2
AUC based on 50 MI*	68.3% (95%R CI: 63.3%–73.1%)	97.5% (95%R CI: 96.0%–98.9%)
Classification Accuracy based on 50 MI*	65.5% (95% CI: 56.8%–74.1%)	93.0% (95% CI: 88.4%–97.7%)
PPV*	64.8% (95% CI: 52.9%–76.6%)	93.1% (95% CI: 86.6%–99.6%)
NPV*	66.3% (95% CI: 53.6%–79.0%)	93.0% (95% CI: 86.4%–99.6%)
Sensitivity*	69.2% (95% CI: 57.4%–81.1%)	93.1% (95% CI: 86.6%–99.6%)
Specificity*	61.6% (95% CI: 49.0%–74.2%)	92.9% (95% CI: 86.3%–99.5%)

*NC* non-Cancer, *CA* Cancer, *MI* Multiple Imputations, *ROC* Receiver Operating Characteristic, *AUC* Area Under Curve, *CI* Confidence Interval, *PPV* Positive Predictive Value, *NPV* Negative Predictive Value. *P-value between two results was <0.0001.

## Discussion

The purpose of these studies was to optimize LCRT classifier performance prior to clinical validation in the multi-site prospective blinded LCRT cohort trial [30]. Here we report that an LCRT classifier comprising 12 GM genes plus demographic factors has significantly higher accuracy than demographic factors alone for classifying CA from NC individuals. The features comprised by this classifier substantially overlap those in the previously reported LCRT [14]. That said, the classifier presented here represents a new model that must be confirmed in an independent cohort. Toward this goal, the LCRT prospective trial is expected to be completed in 2017–18. The trial design, population characteristics, biospecimen repository, and safety of bronchoscopy to collect bio-specimens were published recently [30]. The selected transcript abundance features will be measured in the LCRT cohort using the high throughput targeted NGS method that was developed in this laboratory [29, 32] and used in the study reported here.

We hypothesize that the lung cancer-associated NBEC gene expression pattern comprised by the classifier represents a genetically determined GM gene protective capacity responsible for inherited predisposition. The degree to which the genetically determined pattern interacts with environmental effects (i.e., cigarette smoking) to determine lung cancer risk was difficult to determine in this case control study because both cases and controls were enriched for heavy smokers. We will test the hypothesis that the classifier represents inherited predisposition in the blinded, prospective LCRT cohort trial [30]. Specifically, the LCRT classifier will be measured in all 384 enrolled subjects, with LCRT classifier measurement conducted blinded to outcome. After 7–8 years of follow-up (2018–2019) we expect >20 incidental lung cancers which is sufficient power to identify a > 2-fold increased risk [30]. Because the classifier reported here detected 33-fold increased risk (95% CI = 12, 102) the LCRT trial will be strongly powered to detect classifier effect. Because all subjects enrolled in the LCRT trial were confirmed not to have lung cancer at the time of study entry, validation will support the hypothesis that the classifier expression pattern is a cause of, rather than a response to, lung cancer.

The hypothesis that the association of LCRT value is based in part on inherited predisposition will be supported by validation of the classifier in the prospective LCRT study but will require additional experimental and epidemiologic confirmation through studies similar to those recently reported [18–21]. In prior experimental studies we reported that germ-line variation is responsible for inter-individual variation in regulation of the key nucleotide excision repair gene ERCC5, one of the genes in the classifier reported here, and a key transcription regulator of GM genes, CEBPG [19–21]. Additional studies will be designed to identify DNA variants that contribute to regulation of other genes comprised by the classifier reported here.

The intended use of the LCRT biomarker is to better stratify individuals for annual LDCT lung cancer screening. Specifically, the goal is to identify individuals who meet current eligibility criteria for annual LDCT screening but have such low risk based on LCRT biomarker measurement that they can be safely advised to opt out of screening. The high specificity of the biomarker presented here when threshold is set for high sensitivity supports the hypothesis that this biomarker will have clinical utility for the intended use. Given that 5–10 million are estimated to be eligible for screening at an estimated cost of $2–4 billion/year [10], if only the 10% with lowest risk based on LCRT opt against screening this will reduce the cost of screening by hundreds of millions of dollars/year and reduce unnecessary tests and procedures resulting from false positive screening results. Implementation for this purpose likely will depend on results of a well-designed prospective randomized clinical trial. That said, due to costs of the procedure, it is likely that this test initially would be restricted to individuals who are having a standard of care bronchoscopy for some other purpose, including investigation for possible lung cancer. For those who are determined not to have lung cancer following bronchoscopy, a negative LCRT biomarker value would provide evidence to support withdrawal from annual lung cancer screening after completion of the active lung cancer work up.

A second possible use of this biomarker may be to guide intensity of diagnostic testing in the setting of imaging abnormalities that raise the question of lung cancer, as described for other recently reported tests [27, 28]. For example, those subjects with LCRT biomarker value below the threshold might be safely monitored by imaging or other non-invasive methods without having to undergo invasive diagnostic tests and/or surgery.

There are ongoing efforts to identify the demographic criteria that best stratify subjects in the effort to optimize outcomes, beyond the current LDCT screening criteria (age 55–80, >30 pack-years) [4, 40–43]. Thus, it is likely that the best methods to optimally stratify subjects for inclusion in LDCT screening will combine the LCRT classifier combining genetic and demographic factors reported here modified to accommodate recently reported demographic criteria [3, 4, 6, 44]. A decision regarding the fraction that would have low enough risk to safely opt out of screening might depend on many other factors, including occupational exposure, family history, and confidence limits associated with the biomarker after the validation study.

Of the twelve genes represented in the classifier reported here, six (E2F1, ERCC4, ERCC5, GPX1, GSTP1, and XRCC1) were in the previously reported classifier [14]. For ERCC5, two separate assays that targeted different alternative transcripts contributed to accuracy of the classifier reported here. Transcript abundance values measured by the two different assays were not correlated, indicating different regulation control and, therefore, independent association with lung cancer risk.

While CEBPG was not included in the optimized classifier it is highly correlated with several of the genes comprised by the classifier reported here. Further, we previously reported that a set of AO, DNAR, and CCC genes containing CEBPG recognition sites display transcript abundance correlation with CEBPG, consistent with regulation by CEBPG, and also display lung cancer-associated expression patterns in NBEC [15]. There is increasing evidence that CEBPG plays a key role in regulating a variety of stress response pathways, including those represented by the LCRT biomarker reported here [45]. For example, this laboratory experimentally confirmed that CEBPG up-regulates ERCC5 [21], and identified ERCC5 SNPs that may contribute to inter-individual variation in regulation of ERCC5 [20, 46]. Thus, it is reasonable to speculate that inter-individual variation in CEBPG expression combined with variation in ERCC5 *cis*-regulatory SNPs contributes to lung cancer risk determination through effect on regulation of one or both of the alternative ERCC5 transcripts.

The six new genes in the classifier reported here include key GM genes CDKN1A, ERCC1, KEAP1, RB1, TP53 and TP63. CDKN1A is a marker for lung cancer and uncontrolled cell proliferation [47, 48]. ERCC1 is a key DNA repair gene that interacts closely with ERCC5 in nucleotide excision repair [49]. KEAP1 (Kelch-like ECH-associated protein 1) is a key regulator of antioxidant defense. KEAP1 inhibits nuclear factor erythroid 2-related 2 (NEF2L2; also named NRF2)-induced cytoprotection. TP53 is involved in cell-cycle checkpoint, DNA repair, senescence, and apoptosis functions [50]. KEAP1, RB1, and TP53 are frequently mutated in squamous lung cancers [51–54]. TP63 expression is a marker for NBEC stem cells [55] and squamous lung cancer [56], and TP63 is frequently mutated in squamous lung cancer [53].

The targeted NGS method used in this study has excellent control for analytical variation, along with relatively high throughput and low cost, and is highly reproducible across platforms, users, and experiments [29, 32]. These characteristics will facilitate analysis in the prospective cohort LCRT study. Prior to analyzing LCRT prospective trial samples we will take steps to minimize missing values. Specifically, for this study, we used only one ISM mixture in which 600 copies of IS and 6000 copies of ACTB IS were in each assay. However, for some highly expressed genes (e.g. GSTP1), the high NT value resulted in missing values due to low sequencing counts for the IS, and for some lowly expressed genes (e.g. E2F1) the low NT value resulted in missing values due to low sequencing counts for the NT. Thus, for the prospective trial we will minimize missing values by measuring each sample using two ISM, with different target IS concentrations.

The ethnic make-up of the cohort used in the study reported here to optimize the LCRT classifier is similar to the make-up of the prospective LCRT cohort that will be used for clinical validation [30]. Due to the relative homogeneity of these populations comprising predominantly non-Hispanic white individuals, assessment of clinical utility in other populations will require additional validation studies.

## Conclusions

In summary, the optimized LCRT biomarker is ready for validation testing in specimens from the prospective LCRT cohort (ClinicalTrials.gov NCT 01130285) [30]. It is expected that a sufficient number of incidental cancers will be observed to reach power by 2018–2019. In addition, we will continue studies to identify *cis*-rSNPs responsible for inter-individual variation in NBEC regulation of genes comprised by the classifier reported here through integration of databases for NBEC transcript abundance and eQTL in lung [57] and experimental confirmation.

## Additional file

Additional file 1: Table S1. List of assays, SNP site, primer and internal standard sequences, demographics, and transcript abundance (target gene molecules/10^6^ ACTB molecules). Bold: Selected features. (XLSX 10^3^ kb)

## Abbreviations

AO: Antioxidant
AUC: area under the curve
CA: lung cancer
CAT: correlation adjusted t.
CCC: cell cycle control
CV: coefficient of variation
DNAR: DNA repair
GM: genome maintenance
IRB: Institutional Review Board
IS: internal standard
ISM: internal standard mixture
ISR: integrated stress response
LCRT: lung cancer risk test
LDA: linear discriminant analysis
LDCT: low dose CT
MICE: Multivariate Imputation by Chained Equations
NBEC: normal bronchial epithelial cell
GS: next generation sequencing
NLST: National Lung Screening Trial
NT: native template
PMM: Predictive Mean Matching
ROC: Receiver Operator Characteristic
USPSTF: United States Preventive Safety Task Force
